# Neonatal mesenteric cyst in a 5-day-old patient: a case report

**DOI:** 10.11604/pamj.2024.48.46.43778

**Published:** 2024-06-05

**Authors:** Seth Jotham, Alicia Massenga, Geofrey Giiti, Ally Rashid, Erasto Wambura, Fabian Mghanga

**Affiliations:** 1 Department of Surgery, St. Francis University College of Health and Allied Sciences, Ifakara, Tanzania,; 2 Department of Surgery, Catholic University of Health and Allied Sciences, Mwanza, Tanzania,; 3 Department of Clinical Medicine, Tanzania Training Centre for International Health, P.O. Box 39 Ifakara, Kilombero, Tanzania

**Keywords:** Neonate, mesenteric cyst, segmental resection, case report

## Abstract

Mesenteric cysts have been documented as a rare occurrence in children. They are mostly renowned to be benign intra-abdominal tumors with no known etiology. The symptoms are non-specific ranging from being asymptomatic to an acute abdomen. Most of the diagnoses are made below the age of 10 years with devoid of reports for the early neonatal occurrences. We report a case of an early neonatal mesenteric cyst in a 5-day-old female patient who presented with signs of intestinal obstruction markedly by abdominal distension, vomiting, and absolute constipation. Abdominal X-ray showed evidence of small bowel obstruction while abdominal ultrasound and computed tomography (CT) scan were used to reach the diagnosis of a mesenteric cyst, all laboratory baseline investigations were within a normal range. On laparotomy a 12 by 13 cm cyst that was firmly adhering to the proximal ileal wall was meticulously dissected, complete cystectomy was done with no segmental resection. Histopathologically there were no signs of malignancy and the patient successfully recovered with no signs of recurrences after being followed for a year and a half. Being a rare case in the early neonatal period with unspecific presentations; mesenteric cyst should be considered as one of the diagnoses best to be managed by surgical excision to prevent recurrences.

## Introduction

Mesenteric cysts are commonly known to be the benign masses growing along the mesenteries. Though said to occur in any age group with about 1/3 of cases occurring in the pediatric population below the age of 15 years, it has been rarely reported during the early neonatal period [1]. The condition is set to occur along any part of the gastrointestinal mesentery from the duodenum to the rectum, however about 60% of the cysts are said to occur along the small bowel mesentery, 24% in large bowel mesentery, 14.5% in retro peritoneum with about 1.5% having an indefinite occurrence [2]. Its exact etiology is yet to be ascertained but the common theory is explained by the failure of the mesenteric lymph nodes to communicate with the rest of the lymphatic vessel or the venous system due to blockage that can be caused either by trauma, infection, or neoplasm [2,3]. The mesenteric cyst has no peculiar presentation but abdominal pain, constipation, nausea, and vomiting have been well pronounced and can also point towards any other differential [4,5].

Radiological investigations such as ultrasound remain the standard of diagnosis and in most cases; a contrast CT scan is needed to qualify the mass´s origin, orientation, and surrounding structures, rarely is magnetic resonant imaging required [3,6]. Minimal-invasive surgery is the recommended surgical approach however when not feasible; an open approach should be implied to achieve a complete excision [7,8]. In neonates, mesenteric cyst still poses a diagnostic challenge as they may mimic other conditions like lymphangioma intestinal duplication and even ovarian cysts in females. We then present a case of a 5-day-old patient diagnosed with an ileal mesenteric cyst at Bugando Medical Centre (BMC), a tertiary and teaching institution serving the Northwestern Zone of Tanzania along the coasts of Lake Victoria.

## Patient and observation

**Patient information:** a 5-day-old female patient was brought to the BMC pediatric surgical department as a transfer from the pediatric medical department. She presented with an abdominal distension post-delivery which markedly increased over the five-day course. It was associated with vomiting and constipation which was noted on day three of life. She is the second born from a 35-year-old mother who reports no such occurrence on the first 3-year-old baby. There was no remarkable intervention done prior to being brought to the BMC pediatric surgical department.

**Clinical findings:** on examination, the baby was alert with no signs of dehydration. She was neither pale nor jaundiced with stable vital signs. Her abdomen was markedly distended giving a dull percussion note over the protuberant, the rest being hyper tympanic. A palpated mass with a cystic consistency was felt, and the bowel sounds were exaggerated with a normal digital rectal examination finding. Other systems were remarkably normal.

**Timeline:** the patient was received in the pediatric surgical department on day 5 of life after she was noticed to have significant abdominal distension accompanied by constipation and vomiting. She was operated on the next day which was her 6^th^ day of life and discharged home 6 days later post-surgical intervention.

**Figure 1:**
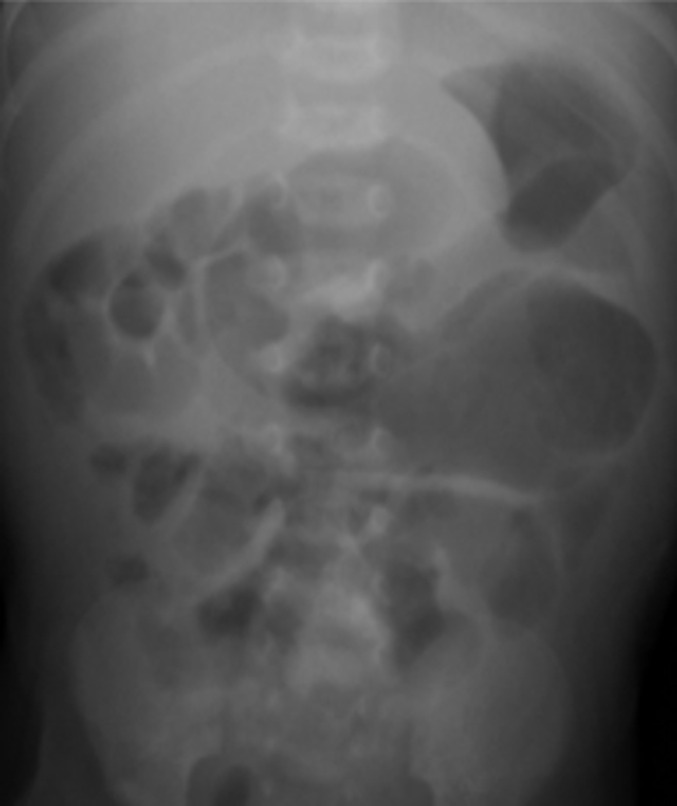
plane abdominal X-ray with a nonspecific dilatation of bowel loops

**Figure 2:**
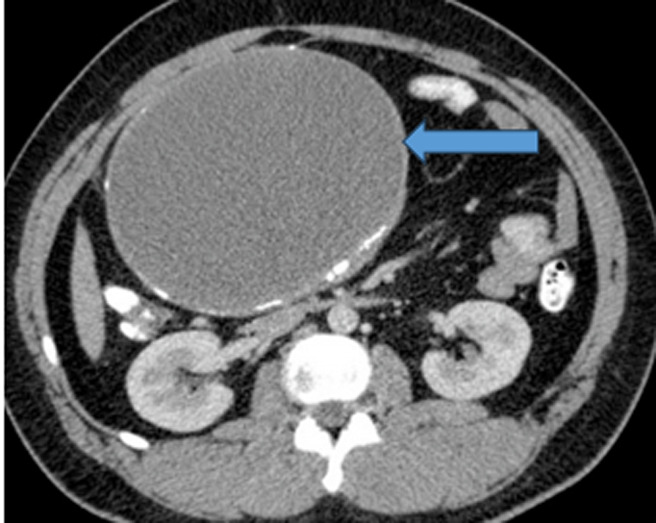
large intra-abdominal homogenous cystic mass (blue arrow) within enhancing walls in keeping with a mesenteric cyst

**Diagnostic assessment:** abdominal X-ray showed nonspecific dilatation of bowel loops (Figure 1), and abdominal ultrasound (USS) showed an anechoic diffuse lesion more suggestive of a cyst. A contrast CT scan showed a large intra-abdominal homogeneously mass within enhancing walls consistence with a mesenteric cyst that measured about 11 by 13.2 cm (Figure 2). Alpha-fetoprotein (AFP), Beta human chorionic gonadotropin hormone (β-hCG), and other baseline laboratory investigations were within normal ranges.

**Figure 3:**
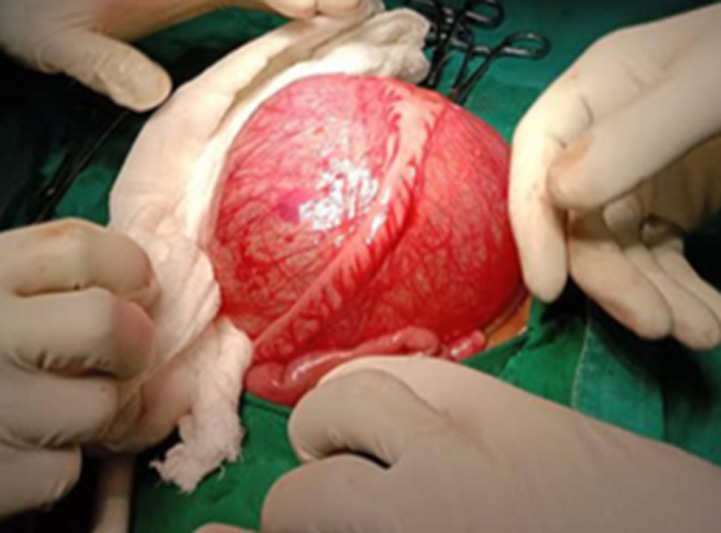
intra-operatively, an Ileal mesenteric cyst closely and firmly adherent to the proximal ileum

**Therapeutic interventions:** assent was sought from both parents post-counseling and the patient was prepared for surgery without any delay. On laparotomy, a unilocular cystic intraperitoneal mass of about 12 by 13 cm was found, it originated from the mesentery of the ileum which was closely and firmly adherent to the proximal ileum (Figure 3). A meticulous dissection was done and a complete cystectomy was achieved with no segmental resection. The abdomen was closed in layers and the baby recovered well from the general anesthesia.

**Follow-up and outcomes:** she was taken to the neonatal intensive care unit following her surgery, where she was monitored for a whole day before being transferred to the normal ward for the other regular post-operative care; six days after the procedure, she was sent home. She had a benign mesenteric cyst as later evidenced by the histological results and hence clinical follow-up was done successfully with serial evaluation for a year and a half with no complications or indicators of recurrence.

**Patient perspective:** though her parents had great concern at the beginning, they were pleased with the facility's services and intervention, and the 1.5-year follow-up period gave them greater confidence and comfort simply by witnessing their second child grow normally as their first.

**Patient consent:** written assent was obtained from the patient's parents for publication of this case report with its accompanying images. This case report was approved by the Joint CUHAS/BMC Research, Ethics and Review Committee.

## Discussion

In the early 16^th^ century, the Italian anatomist Benevieni first reported a mesenteric cyst after performing an autopsy on an 8-year-old girl [2]. It is commonly seen to be benign with different fluid appearances depending on the level of the gastrointestinal mesentery it has occurred; for instance, at the level of the small intestine and the colon it will be serous, and those at the level of the duodenum it presents as chylous. Multiple cases have been reported during the second decade of life and in pediatric groups of 1-10 years of age and exceptionally in those with less than 1 year. The condition is said to be rare in pediatric groups having an incidence of 1: 200000-250000 pediatric admissions with a mean age of 5 years [9]. Our case presents a mesenteric cyst diagnosed at an early neonatal period in a 5-day-old female patient.

The ileal mesentery is the commonest anatomical site affected followed by the colonic mesentery and depending on the level of its occurrence; fluid encased can either be chylous for the duodenal or serous for the ileal-colonic mesentery [2,3]. This was observed to be the case in our patient as she had an ileal mesenteric cyst which was serous tallying with existing evidence.

Up to date there is no known specific way expected for a patient with a mesenteric cyst to present. This becomes more vogue when it comes to the pediatric group factoring out the subjective symptoms like pain and nausea [4,5]. Our patient, first presented with abdominal distension initially thought to be of a functional cause, it clinically became a concern when she presented with absolute constipation and vomiting on day three of life which warranted her further investigations without a clue of the typical diagnosis. Yoon J *et al*. report the symptoms to be variable and non-specific where 82% of his patients presented with abdominal pain followed by nausea, vomiting, constipation, and the least diarrhea. Jane Jye *et al*. [3] patients mostly presented with abdominal distension and a palpable abdominal mass as observed in Cochran *et al*. report and literature review [10]. In other studies, the mesenteric cyst was an incidental finding as patients presented with neither of the aforementioned symptoms. The presentation in our patient should raise an alarm in any symptomatic obstruction and consider mesenteric cyst as among the differentials during an early neonatal period.

Ultrasound scan (USS), computed tomography (CT) scan, and magnetic resonance imaging (MRI) have proven to be useful in making the diagnosis. The sensitivity of an USS is unquestionable, however; its specificity is challenged by arrays of other intra-abdominal cysts having the same radiological characteristics with significant interobserver variations [6]. In our patient, USS was done first suggesting an intra-abdominal cyst followed by a contrasted abdominal pelvic CT scan which was able to qualify the type of cyst based on its mesenteric origin along with other features. We therefore recommend the utilization of both USS as soon as the cyst is clinically suspected and CT scan for diagnostic accuracy. After treatment, USS should remain a radiological investigation of choice in making follow-up and reserving the CT scan and MRI for the accuracy of the initial diagnosis.

Surgical complete excision of a cyst is a gold standard approach in management, segmental resection and primary anastomosis should be applied based on the individual patient´s intraoperative findings [7,8]. We successfully managed our patient with a meticulous complete excision without segmental resection, and the patient fared well with no recurrence noted during the complete follow-up period.

## Conclusion

Despite being rare with unspecific presentations, early mesenteric cyst is not uncommon, and a complete surgical excision with or without segmental resection remains to be the gold standard modality of management.
